# A Nurses' Club in Dublin

**Published:** 1920-02-07

**Authors:** 


### A NURSES' CLUB IN DUBLIN.

The College of Nursing in Ireland has made a long forward stride. It has provided the nurses of the country, College members and others, with a first-class club, social and residential. The premises, as I mentioned in a previous issue, are well appointed and commodious, situate in Fitzwilliam Square, one of the best residential parts of Dublin. A few months ago a small gathering assembled in one of the rooms of this house to consider how a sum of £2,000 might be obtained to equip and start the club. The task seemed a difficult one to look forward to, as the public have been pretty well plucked. However, on Wednesday of last week, when Lady Powerscourt declared the club open, Sir John Lumsden made the pleasing announcement that the Red Cross had provided one moiety and the Earl of Iveagh had generously contributed the other. It is interesting to know that Sir Cooper Perry was instrumental in bringing this deserving cause under Lord Iveagh's notice and obtaining so handsome and practical a co-operation, one which has really ensured success from the start. The Red Cross gift was conditional on an equal sum being otherwise subscribed. There are other oddments still wanting, such as pictures and additions to the furniture, but these will be quickly forthcoming, as fêtes and entertainments are under way for the purpose.

### The Opening Ceremony.

Ireland is a land of schisms and factions and the nursing world corresponds. Hence it was particularly interesting to note that the distinguished speakers and the crowded audience represented all the talents. Sir John Lumsden, K.B.E., opened the proceedings, and in a very happy speech recounted the various steps by which the College had advanced from nowhere to here. The Lord Chief Justice of Ireland followed, and accentuated what Sir John Lumsden announced, that it was to be in every respect a Home Rule Club, to be managed exclusively by nurses who could make of it what they chose. The "mere men" who had helped in its establishment would retire as soon as it was on its feet. Then a prominent Roman Catholic clergyman, the Rev. Father Hatton, gave the club his blessing, and expressed his hearty approval of the non-sectarian spirit in which it had been established. Father Hatton's presence and speech were an asset of great value to the proceedings, putting it beyond yea or nay that in this institution at all events neither politics nor creed will count. The uniform of the nurse will be the badge of membership. If the management follow the lead of the founders, there will be a complete absence of snobbishness, cliques, or factions.

The announcement that Miss Rundle had travelled from London in order to be present was warmly applauded. Miss Rundle had crossed the Irish Sea the day previous in a hurricane, and she testified with feeling on her arrival that she found coming to the hostel like coming home. All her wants had been anticipated. Miss Rundle dwelt on the progress that the College had made since her previous visit three years ago. I can quite realise her feelings, for I was present when Miss Rundle and Miss Cox-Davies came as the College pioneers in those early days. It was not over the Irish Sea that the hurricane then blew. The Chairman who presided struck the first discordant note, and the chorus that followed was worthy of Donnybrook Fair. The Englishwomen were heckled and abused without mercy. Irish chivalry and Irish hospitality were conspicuous by their absence, and a College of Nursing establishment in Ireland seemed a forlorn hope. But, true to their traditions, these ladies accepted the situation with a smile. Every question was answered with a candour and a simplicity that disarmed criticism. With consummate tact, worthy of a great diplomatist, these ladies let the tempest of abuse pass, and confined their attention to actual facts. On this occasion Miss Rundle displayed the same big mind, and did not forget to congratulate the advocates of State Registration in Ireland on the victory they had achieved.

A particularly felicitous speech was made by Sir Andrew Horne, one of Dublin's most distinguished physicians, and a stalwart supporter of the College since its inception in Ireland. I do not know Sir Andrew's age. I should size him up as about fifty-five, but he described himself as one of the oldest doctors in Dublin, and he looked back to a time when there were no trained nurses in the city. Good old women of seventy or thereabouts were in charge of large wards full of typhus patients. Verily the scene is changed. Sir Andrew Horne is a College champion without reserve. Miss Rundle was good enough to express her envy of Ireland in having established a hostel, which, so far, London has not accomplished. Sir Andrew Horne gave his experience, and testified to the warm hospitality which he had experienced at the College of Nursing headquarters in London.

There was just one untoward event in the proceedings, and it is worth chronicling. When the speechmaking ended a lady in the audience complained that "Irish" nurses who had not seen their way to join the "English" College were to be penalised by having to pay half a guinea for membership, while College members pay only five shillings.

Of course, the answer is obvious. College members have already paid a guinea subscription, and, instead of penalising other nurses, they are extending a privilege to them. If they kept the Club exclusively in their hands there would be no ground for complaint. This is a trivial matter. But greater attention ought to be given to explaining that the College is not "English," but British and Imperial, whilst Ireland is likewise British. Inaccurate nomenclature is often responsible for grave misunderstanding. Long before the Christian era Aristotle referred to "the British Islands" as consisting of Albion and Ierne. They still remain the British Islands.

There is one name that was not mentioned at the opening meeting of the Club—that of Miss Matheson, Secretary to the College. With due respect to all the others, I think it is simple truth that but for Miss Matheson there would be no club. She pioneered the movement and kept it in the foreground from the beginning. It is now in charge of an exceedingly efficient Secretary, and the outlook is bright and full of promise. IERNE.

